# Cardiopulmonary exercise test in patients with refractory angina: functional and ischemic evaluation

**DOI:** 10.1016/j.clinsp.2021.100003

**Published:** 2022-02-05

**Authors:** Camila R.A. de Assumpção, Danilo M.L. do Prado, Camila P. Jordão, Luciana O.C. Dourado, Marcelo L.C. Vieira, Carla G. de S.P. Montenegro, Carlos E. Negrão, Luís H.W. Gowdak, Luciana D.N.J. De Matos

**Affiliations:** a Instituto do Coração (InCor), Hospital das Clínicas HCFMUSP, Faculdade de Medicina, Universidade de São Paulo, São Paulo, SP, Brazil; b Escola de Educação Fisica e Esporte, Universidade de São Paulo, São Paulo, SP, Brazil; c ULTRA Exercise Physiology Laboratory, São Paulo, SP, Brazil; d Hospital Israelita Albert Einstein, São Paulo, SP, Brazil

**Keywords:** Cardiopulmonary exercise testing, Oxygen uptake efficiency slope, Exercise capacity, Oxygen pulse, Myocardial ischemia

## Abstract

**Objectives:**

Refractory angina (RA) is a chronic condition clinically characterized by low effort tolerance; therefore, physical stress testing is not usually requested for these patients. Cardiopulmonary exercise testing (CPET) is considered a gold standard examination for functional capacity evaluation, even in submaximal tests, and it has gained great prominence in detecting ischemia. The authors aimed to determine cardiorespiratory capacity by using the oxygen consumption efficiency slope (OUES) in patients with refractory angina. The authors also studied the O_2_ pulse response by CPET and the association of ischemic changes with contractile modifications by exercise stress echocardiography (ESE).

**Methods:**

Thirty-one patients of both sexes, aged 45 to 75 years, with symptomatic (Canadian Cardiovascular Society class II to IV) angina who underwent CPET on a treadmill and exercise stress echocardiography on a lower limb cycle ergometer were studied. ClinicalTrials.gov: NCT03218891.

**Results:**

The patients had low cardiorespiratory capacity (OUES of 1.74 ± 0.4 L/min; 63.9±14.7% of predicted), and 77% of patients had a flattening or drop in O_2_ pulse response. There was a direct association between Heart Rate (HR) at the onset of myocardial ischemia detected by ESE and HR at the onset of flattening or drop in oxygen pulse response detected by CPET (R = 0.48; p = 0.019).

**Conclusion:**

Patients with refractory angina demonstrate low cardiorespiratory capacity. CPET shows good sensitivity for detecting abnormal cardiovascular response in these patients with a significant relationship between flattening O_2_ pulse response during CEPT and contractile alterations detected by exercise stress echocardiography.

## Highlights

• OUES analysis is useful for assessing functional capacity in refractory angina.
• O_2_ pulse curve is correlated with contractile alterations in exercise echocardiogram.
• Cardiopulmonary exercise test is useful toll in patients with refractory angina.

## Introduction

Refractory angina (RA) is a chronic condition clinically characterized by low effort tolerance, which has a tremendous impact on daily activities, physical capacity, and quality of life.^1^^,^^2^ Functional capacity is an important marker of prognosis in populations with and without cardiovascular disease.^3^^,^^4^

Because of limited exercise tolerance, physical stress testing is not usually requested for RA patients. Cardiopulmonary exercise testing (CPET) is considered a gold standard examination for functional capacity evaluation. This test is recognized as a valuable diagnostic and prognostic tool for the evaluation of cardiorespiratory diseases, including coronary artery disease (CAD).^5^^,^^6^ Parameters obtained in the CPET, such as peak oxygen consumption (peak VO_2_) and pulmonary ventilation and carbon dioxide production slope (VE/VCO_2_ slope), are widely used as prognostic indicators in clinical cardiology. In addition, CPET is very useful for determining pathophysiological causes of exercise limitation.^4^^,^^5^ Oxygen consumption efficiency slope (OUES) has been shown to be useful in the evaluation of cardiorespiratory capacity in heart disease.^7^^,^^8^ In addition, the OUES has the advantage of not requiring a maximum test.^9^^,^^10^ Therefore, the OUES is a useful tool for functional capacity assessment in patients with clinical limitations, as those with RA.

CPET has gained great prominence in detecting ischemia.^11^^,^^12^ The oxygen pulse (O_2_ pulse) defined by VO_2_ per heart rate (HR) has been used to assess myocardial ischemia and as a surrogate indicator of left ventricular stroke volume during exercise.^12^ Previous studies^11^, ^12^, ^13^, ^14^ have suggested that the early plateau or decline of the O_2_ pulse curve can be more sensitive for the diagnosis of ischemia compared with ST-segment depression detected by ECG exercise testing. Furthermore, the analysis of VO_2_ increase as a function of work rate (ΔVO_2_/ΔWR) associated with O_2_ pulse curve has greater accuracy for the diagnosis of myocardial ischemia by CPET.^11^^,^^13^

The aims of this study were (1) to determine cardiorespiratory capacity by OUES in RA patients, (2) to observe the O_2_ pulse response by CPET, and (3) to investigate a possible association between the ischemic changes reflected by flattening or drop in O_2_ pulse response in CPET and contractile modifications in the exercise stress echocardiography (ESE), a gold standard exam to assess ischemia.

## Methods

### Study design

This was a cross-sectional clinical study performed in patients with refractory angina in a tertiary university hospital. All patients were participating in the study “Cardiac rehabilitation in patients with refractory angina” (FAPESP n° 201400345-0), approved by the ethics and research committee of the Clinical Hospital of the Medical School of São Paulo University (CAAE: 24308213.7.0000.0068) and registered at clinicaltrials.gov (NCT03218891). Investigations followed the Declaration of Helsinki. All patients provided written informed consent.

### Study population

Patients of both sexes aged 45 to 75 years, with symptomatic angina, Canadian Cardiovascular Society class (CCS) II to IV, at least three months of duration on optimal medical therapy, not eligible for surgical or percutaneous myocardial revascularization procedures, and exercise stress echocardiograms positive for ischemia were enrolled in the study. Exclusion criteria were (1) permanent pacemakers or implantable cardiac defibrillators; (2) patients with non-sinus rhythm; (3) history of recent (< 3 months) acute coronary syndrome or myocardial revascularization (percutaneous or surgical); (4) functional impairment caused by any clinical conditions preventing exercise; (5) left ventricular ejection fraction (LVEF) < 45%, and (6) patients who did not achieve at least 180 seconds of effort on CPET.

### Cardiopulmonary exercise test

The CPET was performed on a motorized treadmill (T2100 Model, GE Healthcare, USA) and ergospirometer (SensorMedics – VmaxAnalyzer Assembly, Encore 29S, USA), using a graded exercise protocol (Balke 2.5 mph). The exercise workload (speed and/or slope) was increased by one metabolic equivalent (MET) (i.e., 3.5 mL/[kg. min]) every minute until the interruption criteria were met, according to the Guidelines of the Brazilian Society of Cardiology on exercise testing.^15^ HR was continuously recorded using a 12-lead electrocardiogram (Ergo PC, Micromed, Brazil). CPET was performed following the guidelines, as well as the criteria for defining maximal effort and determination of the anaerobic threshold (VAT).^3^, ^4^, ^5^, ^6^^,^^16^ To define a maximum pain level during a CPET and interrupt the test, if necessary, a numeric rating pain scale was used.^17^

### Oxygen uptake efficiency slope (OUES)

The OUES was assessed based on the respiratory data during exercise by calculating the slope of the linear relationship between VO_2_ (y-axis) and the logarithm of VE (x-axis) using single regression analysis. Before inclusion in the regression analysis, respiratory data were averaged every 30s from the beginning of the second minute of exercise until evident exhaustion.^18^^,^^19^ The OUES was calculated from data taken from 100% of the exercise test duration. The percent-predicted OUES value was calculated using the equation proposed by Hollenberg et al.^19^

### ΔVO_2_/ΔWR slope, ΔO_2_pulse/ΔWR, and O_2_ pulse pattern analysis

The oxygen uptake and oxygen pulse as a function of work rate (ΔVO_2_/ΔWR, ΔO_2_pulse/ΔWR, respectively) were calculated using the linear regression model.

In respect to both ΔVO_2_/ΔWR and ΔO_2_ pulse/ΔWR, the authors considered it a normal slope when VO_2_ and O_2_ pulse showed linear response as a function of work rate (Sa) and abnormal slope when these parameters showed loss of linearity or a flattening response (Sb).^11^

The work rate (WR) was calculated based on both the speed and grade of the treadmill and bodyweight of the patient. The WR was determined using the following equation: WR (kg/m^.^m^−1^) = F  ×  S (sine ɵ  ×  D)/60 min, where F = body weight in kg; S = treadmill speed; sine ɵ = sine of the treadmill angle; D = Distance; min = minutes; kg/m^.^m^−1^ = meters per minute relative to body weight.^20^

### Heart rate response

The HR as a function of work rate (ΔHR/ΔWR) and oxygen consumption (ΔHR/ΔVO_2_) were calculated with a linear regression model from the onset of exercise test to 10 seconds preceding VAT (S1) and 10 seconds after VAT to the peak of exercise (S2). The percentage of change in slope ΔHR/ΔWR was calculated as a difference in S2 and S1 divided by S1 and multiplied by 100.^21^

HR response during exercise was also analyzed by the chronotropic reserve (CR), as follows:^14^ (CR) = (Peak HR - Resting HR/ (220 - Age) - Resting HR) × 100.

### Exercise stress echocardiography (ESE)

Two-dimensional echocardiogram evaluation was performed with the Vivid9 device (version 110.x.x, GE Healthcare) and according to the guidelines of the American Society of Echocardiography.^22^ After echocardiography at rest, exercise testing was performed on a lower limb cycle ergometer adapted to the stretcher, with a 45° inclination laterally and 45° horizontally. The workload was progressively increased from 5 to 25 watts every 3 minutes, according to physical capacity for each patient, and echocardiographic analyses were performed during all efforts. The exercise test was interrupted when the patient's reached exhaustion or had pain, hemodynamic or electrocardiographic criteria according to ergometric test guidelines.^22^^,^^23^

In order to assess the left ventricular segmental contractility, a segmental analysis 16-segmental model recommended by the American Society Echocardiography (ASE) was used.^24^ Was defined positive for ischemia on HR and second at the hypokinesis, akineses, or dyskinesis moment.^24^

### Statistical analysis

Data were analyzed using Statistica for Windows (Release 5.0). Continuous variables are expressed as mean ± standard deviation (SD) and categorical variables as percentages. The sample distribution was assessed using the Kolmogorov Smirnov test and characterized by a symmetric distribution. One-way analysis of variance (ANOVA) with repeated measures was performed to test within-group differences for a cardiorespiratory response during graded exercise. When significant differences were detected, Tukey post hoc comparisons were performed. Pearson correlation coefficient was performed to determine the relationship between HR at the onset of flattening O_2_ pulse response and HR related to myocardial ischemia detected by exercise ESE. A p-value less than 0.05 was considered statistically significant.

## Results

### Patients

**Figure 1:**
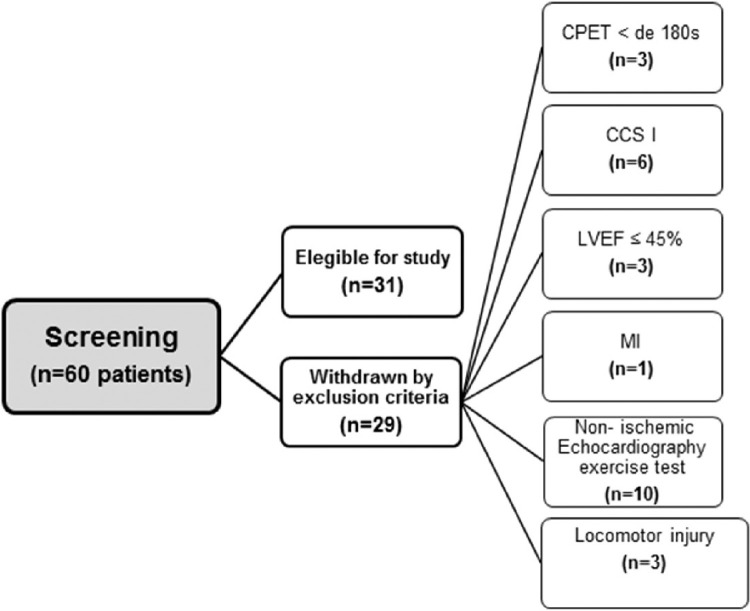
Flowchart of patients. CPET, Cardiopulmonary Exercise Testing; CCS, Canadian Cardiovascular Society Class; LVEF, Left Ventricular Ejection Fraction; MI, Myocardial Infarction.

The flowchart of the patient selection process is illustrated in Figure 1. Of the 60 patients who were screened for participation, 31 met the inclusion criteria. The clinical characteristics and the medication used in the study population are shown in Table 1. The patients had a mean age of 61.3±8.4 years, and 19 (61.3%) were male. Of all patients included, 13 (41.9%) were classified as CCS 2, 7 (22.6%) as CCS 3, and 11 (35.5%) as CCS 4. The number of weekly angina attacks was 7.0 (IQR, 25%–75%; 2.0–21.0), and the weekly use of sublingual nitrate was 1.0 (IQR, 25%–75%; 0.2–7.0).

**Table 1:** Patient characteristics.

Men/Women	19/12
Age (years)	61.3 ± 8.4
**Anthropometric variables**	
Height (m)	1.67 ± 0.10
Weight (kg)	81.4 ± 14.9
BMI (kg/m²)	29.0 ± 3.8
AC (cm)	101.1 ± 10.4
**Hemodynamic variables**	
HR rest (bpm)	61.2 ± 5.9
SBP rest (mmHg)	121.2 ± 16.1
DBP rest (mmHg)	74.4 ± 10.2
**CCS, n (%)**	
2	13 (41.9)
3	7 (22.6)
4	11 (35.5)
**Risk factors, n (%)**	
SAH	25 (80.6)
Dyslipidemia	30 (96.8)
DM	22 (70.9)
Obesity	9 (29)
Smoking	1 (3.2)
FHCAD	21 (67.7)
AMI	25 (80.6)
Sedentary	22 (70.9)
Weekly angina	11.5 ± 11.7
Weekly nitrate	4.5 ± 7.46
**Medications, n (%)**	
BB	31(100)
CCB	28 (90.3)
Clopidogrel	10 (32.2)
Statin	31 (100)
Acetylsalicylic acid	29 (93.5)
Nitrate	23 (74.2)
Trimetazidine	30 (96.8)
Ivabradine	6 (19.3)
ACEIs	15 (48.3)
ARBs	9 (29)
Diuretic	14 (45.1)
Oral antidiabetic	16 (51.6)

Values are means ± SD or n (%). AC, Abdominal Circumference; ACEIs, Angiotensin-Converting Enzyme Inhibitors; AMI, Acute Myocardial Infarction; ARBs, Angiotensin Receptor Blockers; BB, Beta-Blockers; BMI, Body Mass Index; CCB, Calcium Channel Blocker; CCS, Canadian Cardiovascular Society class; DBP, Diastolic Blood Pressure; DM, Diabetes Mellitus; FHCAD, Family History of Coronary Artery Disease; HR, Heart Rate; SAH, Systemic Arterial Hypertension; SBP, Systolic Blood Pressure.

### CPET parameters

**Figure 2:**
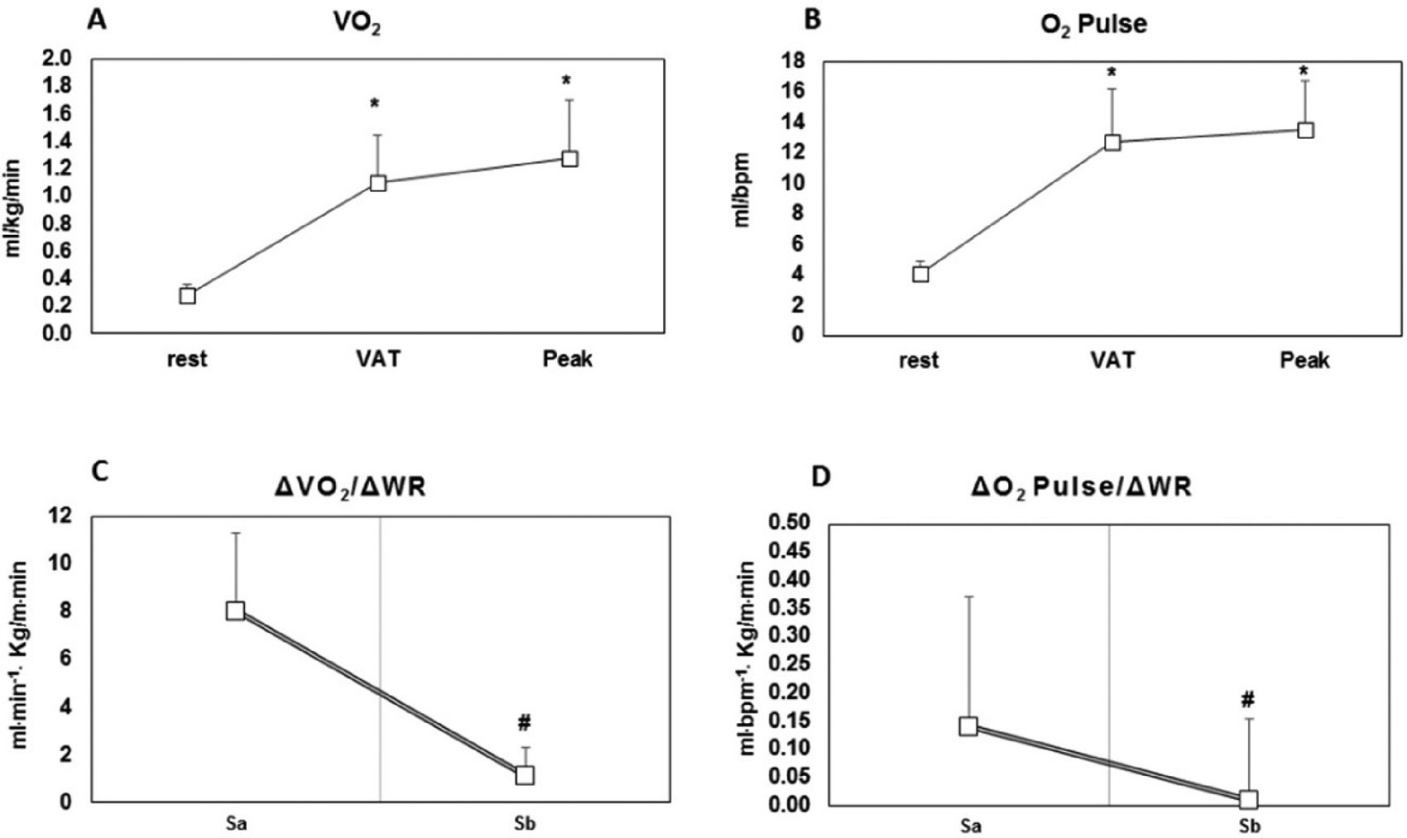
VO_2_ response (panel A); O_2_ pulse response during CPET in patients with refractory angina (panel B); ΔVO_2_/ΔWR slope (panel C); ΔO_2_ pulse/ΔWR (panel D). VO_2_, oxygen consumption; O_2_ pulse, oxygen pulse; VAT, Ventilatory Anaerobic threshold; Sa, linear response of oxygen consumption as a function of work rate; Sb, loss of linearity or a flattening response of oxygen consumption as a function of work rate. * p < 0.05 vs. rest; **#** p < 0.05 vs. Sa.

The CPET results of the patients are shown in Table 2 and Figure 2. The patients demonstrated low cardiorespiratory capacity (Peak VO_2_ = 16.2 ± 3.8 mL/(kg.min); 64.9% ± 17.4 of predicted), and OUES of 1.74±0.4 L/min (63.9% ± 14.7 of predicted). However, the patients had normal aerobic capacity with VO_2_ at the VAT of 12.9 ± 3.0 mL/(kg.min) (52.2%±12.8 of PeakVO_2_ predicted). At peak exercise, the patients showed both RER = 1.0±0.1 and peak HR of 62% of age-predicted.

**Table 2:** Cardiopulmonary exercise test parameters in patients with refractory angina.

**VO_2_**	
Peak (mL/kg.min^−1^)	16.2 ± 3.8
Predicted (%)	64.9 ± 17.4
Patients with early plateau (%)	80
**VAT**	
VO_2_ (mL/kg.min)	12.9 ± 3.0
Peak VO_2_ (%)	79.6 ± 11.7
Peak VO_2_ predicted (%)	52.2 ± 12.8
**O_2_ Pulse**	
Peak (mL/bpm)	13.2 ± 3.7
Peak corrected by the predicted HR (mL/bpm)	8.17 ± 2.7
Predicted (%)	105.6 ± 24.2
Predicted corrected by the predicted HR (%)	65.4 ± 17.9
% Patients with early plateau or decline	77
**OUES**	
L/min	1.74±0.40
mL/(kg.min)	22.3±4.4
Predicted (%)	63.9±14.7
**Chronotropic reserve (%)**	36.0±15.2
**HR peak (bpm)**	98.0±16.0
**Endurance time (s)**	358.1±128.6
**RER peak (units)**	1.0±0.1
**Angina**	
HR (bpm)	89.3±16.4
Pain scale (0–10)	7.0±1.9
**VO_2_/WR (mL^.^m^−1.^kg/m.m^−1^)**	
Sa	8.0±3.3
Sb	1.1±1.2
P	0.001
**O_2_ pulse/WR (mL^.^bpm^−1.^kg/m.m^−1^)**	
Sa	0.14 ± 0.23
Sb	0.01 ± 0.01
P	0.003
**HR/WR (bpm^.^kg/m.m^−1^)**	
S1	0.20 ± 0.12
S2	0.06 ± 0.07
P	0.001
**ΔHR/ΔWR (%)**	-93.6 ± 7.6
**ΔHR/ΔVO_2_**	
S1	16.6 ± 8.4
S2	11.7 ± 12.9
P	0.07

Values are means ± SD or n (%). ECG, Electrocardiogram; HR, Heart Rate; OUES, Oxygen Uptake Efficiency Slope; O_2_ pulse, oxygen pulse; RER, Respiratory Exchange Ratio; s, seconds; VAT, Ventilatory Anaerobic Threshold; VO_2_, Oxygen Consumption.

Regarding VO_2_ response during CPET, the present study's results did not demonstrate differences between VAT and Peak (Fig. 2, panel A; p = 0.19). However, for ∆VO_2_/∆WR, the patients had greater values for Sa than for Sb (8.0 ± 3.3 vs*.* 1.1 ± 1.2 mL^.^m^−1.^kg/m.m^−1^; p = 0.001, respectively) (Table 2 and Fig. 2, panel C). Importantly, the present study's results also showed that 77% of patients had a flattening VO_2_ response during CEPT.

Regarding the O_2_ pulse response, the study's findings did not show differences between VAT and Peak (Fig. 2, panel B, p = 0.47). However, with ∆O_2_pulse/∆WR, the patients had greater values for Sa than for Sb (0.14 ± 0.23 vs. 0.01 ± 0.01 mL^.^bpm^−1.^kg/m.m^−1^; p = 0.003, respectively) (Table 2 and Fig. 2, panel D). In addition, 77% of patients showed both a flattening and a drop in O_2_ pulse response.

Regarding ∆HR/∆WR, the present study's results showed a significant difference between S1 and S2 (0.20 ± 0.12 vs. 0.06±0.07 bpm^.^kg/m.m^−1^; p = 0.001, respectively, Table 2). However, for ΔHR/ΔVO_2,_ the authors did not observe differences between S1 and S2 values (16.6 ± 8.4 vs. 11.7 ± 12.9; p = 0.07, respectively, Table 2). Of note, during CPET, the patients demonstrated a chronotropic index of 36.0 ± 15.2%. In addition, for ΔHR/ΔWR (%) the present study's results showed a negative value of -93.6 ± 7.6%. During CPET, the patients had a pain scale of 7.0±1.9 (Table 2).

### ESE parameters

**Figure 3:**
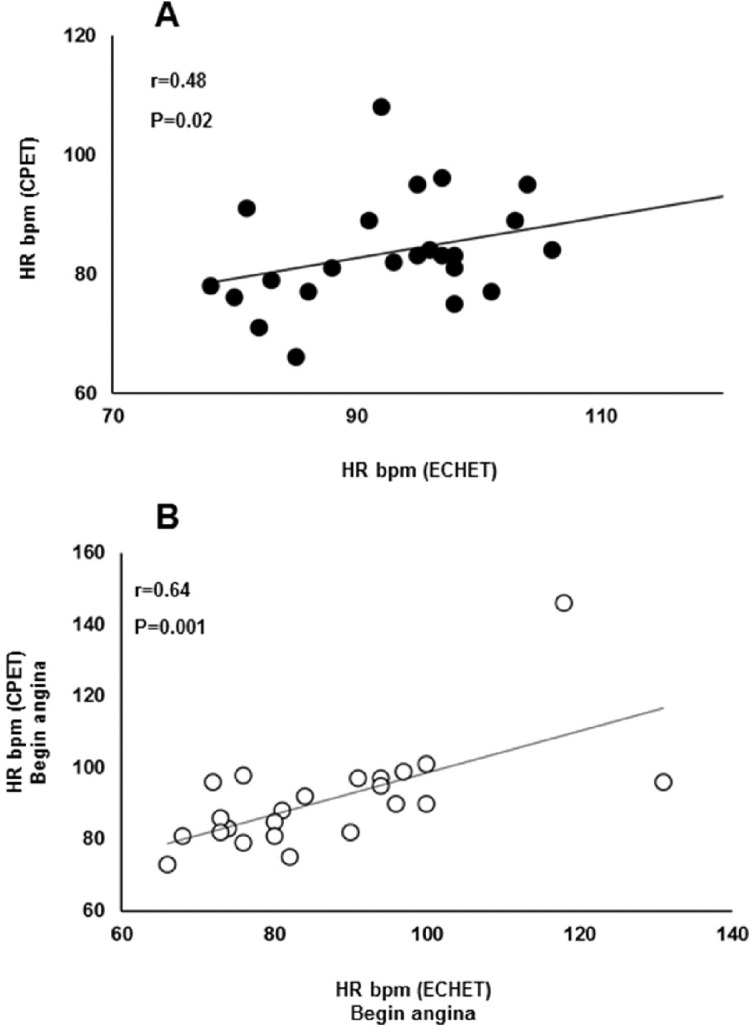
Relationship between HR at onset of flattening oxygen pulse response detected by CPET and ischemic changes with contractile modifications in the ESE (panel A); and HR at onset of angina detected by CPET and ESE. CPET, cardiopulmonary exercise test; ESE, exercise stress echocardiography.

The ESE results are shown in Table 3. At rest, the patients demonstrated a normal LVEF (56.8 ± 6.7%). Contractility score had a significant increase during exercise compared with rest (1.30 ± 0.26 vs. 1.49 ± 0.32; p = 0.001, respectively). Correlation analysis showed a positive association between HR at the onset of myocardial ischemia detected by ESE and CPET (R = 0.48; p = 0.019) (Fig. 3, panel A). In addition, correlation analysis showed a positive association between HR at the onset of angina detected by both exercise testing modalities (R = 0.64; p = 0.001) (Fig. 3, panel B).

**Table 3:** Echocardiography exercise test parameters in patients with refractory angina.

**LVEF (%)**	56.8 ± 6.7
**Score**	
Rest	1.30 ± 0.26
Exercise	1.49 ± 0.32
P	0.001
**HR (bpm)**	
Positive	92.8 ± 12.4
Peak	98.1 ± 14.1
Predicted (%)	61.8 ± 9.1
**Angina**	
HR (bpm)	90.1 ± 14.3
Pain scale (0-10)	5.68 ± 2.89
**Exercise time (s)**	
Positive	225.9 ± 90.6
Total	280.4 ± 111.0

Values are means ± SD; Exercise time, second at the hypokinesis, akineses, dyskinesis moment in seconds; HR, Heart Rate; Positive, HR at the hypokinesis, akineses, dyskinesis moment; LVEF, Left Ventricular Ejection Fraction.

## Discussion

To the authors’ knowledge, this study is the first to specifically investigate cardiorespiratory response during cardiopulmonary exercise testing in patients with RA. The main findings are that (1) RA patients had low cardiorespiratory capacity measured by OUES, (2) Most (77%) of the RA patients had flattening O_2_ pulse response during CPET, and (3) There is a significant association between abnormal O_2_ pulse curve and contractile alterations detected by ESE.

CPET is a highly reliable method for assessing cardiorespiratory capacity and monitoring exercise tolerance in patients with cardiovascular disease.^5^^,^^25^ The OUES provides an objective index for the evaluation of cardiorespiratory function reserve without requiring maximal effort.^18^^,^^26^ In the present study, the authors found a lower cardiorespiratory capacity in patients with RA (OUES corresponding to 63% of age-predicted). The OUES in the studied patients was lower than that observed in healthy subjects (1.74 ± 0.40 vs. 2.55 ± 1.01),^27^ very close to that observed in patients with cardiovascular disease. Davies et al.^8^ found an OUES of 1.6 L/min in patients with heart failure. In the same study, the authors concluded that OUES was a more powerful predictor of mortality than VO_2_ peak and VEVCO_2_ slope.^8^ Similarly, Coeckelberghs et al.^10^ showed that OUES is an important predictor of all-cause and cardiovascular mortality in patients with CAD. Although the present study was not designed to explore the physiological mechanisms underlying OUES, it is possible to suggest factors related to lower OUES in RA patients. The lower cardiorespiratory capacity observed in patients with RA seems to be more related to central than peripheral factors. The patients had values of VO_2_ at VAT within normality limits, suggesting a normal response^5^ to aerobic metabolism during exercise. On the other hand, an important finding observed in the studied patients was the abnormal cardiovascular response during exercise. Notably, the present study's results showed a flattening VO_2_ response during CPET. Moreover, when the authors analyzed both ΔVO_2_/ΔWR and ΔO_2_pulse/ΔWR, the authors found greater values for Sa than for Sb. Therefore, the authors can suggest that this abnormal VO_2_ response observed in RA patients may be related to abnormalities in stroke volume (SV). In this sense, O_2_ pulse is useful as a surrogate indicator of SV changes during exercise in healthy subjects.^5^^,^^21^ Interestingly, a flattening or a decrease in O_2_ pulse curve during physical exercise may be associated with the inability to increase SV to the values necessary to meet the oxygen demands.^5^^,^^11^^,^^12^ Furthermore, the patients demonstrated low inotropic capacity during exercise (i.e., peak O_2_ pulse corrected by the predicted HR = 8.17 ± 3.8 mL/bpm, 65.4 ± 17.4% of predicted).

In light of the above, the question that arises is why patients with RA had an abnormal inotropic response during exercise? This issue is related to the second aim of the present study. The authors suggested a close relationship between the onset of myocardial ischemia and the worsening of left ventricular systolic function during exercise. Previous investigations showed that a delay in VO_2_ response during exercise is related to left ventricular ischemia.^5^^,^^11^, ^12^, ^13^, ^14^ Belardinelli et al.^13^ demonstrated that the association between the ΔVO_2_/ΔWR slope and the O_2_ pulse curve had a sensitivity of 89% for detecting myocardial ischemia by CPET. These researchers evaluated CAD patients by CPET in a cycle-ergometer. They observed a normal ΔVO_2_/ΔWR slope from the beginning of the exercise up to the moment corresponding to the onset of myocardial ischemia (i.e., 9.4 ± 0.5 mL/min/W). Of note, the authors found that the rate of increase in VO_2_ as a function of the work rate was flatter until the peak of exercise (i.e., 3.5 ± 2.0 mL/min/W). In the current study, the RA patients had the same pattern of ΔVO_2_/ΔWR slope. The slope of normal ΔVO_2_/ΔWR (Sa) was greater than that of the flattening response (Sb) (8.0 ± 3.3 vs. 1.1 ± 1.2 mL^.^m^−1.^kg/m^.^m^−1^, respectively). Moreover, the study's results show that during CEPT 80% of patients had a flattening VO_2_ response. The present study analyzed cardiorespiratory response by CPET in a walking protocol on a treadmill. Considering the treadmill as a moving conveyor belt with a variable grade of inclination, the work rate was calculated based on the bodyweight of the patient, speed, and the vertical distance during walking up the incline (i.e., sine of the treadmill angle). Moreover, during the walking protocol, the workload was increased 1 MET per minute with the purpose of a linear increase in VO_2_ during CPET.

Noteworthy, the flattening in O_2_ pulse induced by myocardial ischemia can create a compensatory mechanism by immediately increasing HR to maintain cardiac output to accomplish the adequate oxygen demand during physical exercise.^28^

Previous studies have demonstrated that an increase in ΔHR/ΔWR slope after VAT is associated with atherosclerotic heart disease.^29^ In contrast, the ΔHR/ΔWR slope in the present study showed a blunted HR response after VAT (S1 = 0.20 ± 0.12 vs. S2 = 0.06 ± 0.07 bpm^.^kg/m.m^−1^), with attenuated chronotropic response during exercise, suggesting chronotropic incompetence (CI). Importantly, the patients were receiving beta-blocker therapy. There is solid evidence^30^, ^31^, ^32^ that beta-blockers may attenuate the exercise-induced HR increase during exercise. Previous investigations^33^ have suggested a different CI criterion for patients with heart disease taking beta-blockers. Khan et al.^33^ proposed a value of ≤ 62% for CR in patients taking beta-blockers. In the present investigation, the RA patients had a low chronotropic reserve, which was confirmed by chronotropic index analyses (i.e., 36.0±15.2%). It is well known that CI is related to myocardial perfusion abnormalities analyzed by stress testing with thallium imaging.^34^ Lauer et al.^34^ observed in a cohort of consecutive patients referred for ESE a strong association between CI and echocardiographic findings of myocardial ischemia. Thus, it appears reasonable that RA patients have CI during exercise, despite the use of beta-blockers.

Other interesting information that reinforces the role of ischemia in the present study's findings is the positive association between HR at the onset of myocardial ischemia detected by ESE and CPET. The correlation analysis also showed a significant association between the HR at the beginning of angina detected by both exercise test modalities. Collectively, these findings suggest good sensitivity of the CPET to detect abnormal cardiovascular response during exercise in patients with RA. It is worth noting that 77% of the patients met the criteria for myocardial ischemia detection by CPET. However, electrocardiographic alterations suggestive of myocardial ischemia were found in only 29% of the RA patients.

### Clinical perspectives

CPET can be used as a diagnostic tool for the evaluation of both cardiorespiratory capacity and cardiovascular response during exercise in patients with RA. OUES can be used as an index for functional capacity evaluation in patients with RA. An abrupt flattening of ΔVO_2_/ΔWR and an abnormal O_2_ pulse response during exercise are associated with the onset of myocardial ischemia in RA patients. CPET in the walking treadmill protocol is effective in detecting abnormal cardiovascular response in patients with RA.

### Limitations

This is a descriptive and correlational study, and the authors did not have a control group. Thus, it does not provide conclusive evidence for the physiological mechanisms. Two different ergometers were used (treadmill and cycle ergometer) during CPET and ESE, which may make the interpretation difficult. In addition, the authors found similar HR levels at peak exercise in both exercise tests.

## Conclusion

Patients with RA have OUES during CPET, which is suggestive of low cardiorespiratory capacity in these patients. CPET has good sensitivity for detecting abnormal cardiovascular responses in patients with RA. There is a significant association between flattening O_2_ pulse response during CEPT and contractile alterations detected by ESE.

## Author contributions

All authors contributed to the conception of the work. Assumpção CRA, Prado DML, Jordão CP, Dourado LOC, Vieira MLC, Montenegro CGSP contributed to the data acquisition. Assumpção CRA, Prado DML, Jordão CP, Dourado LOC, Vieira MLC, Montenegro CGSP, Negrão CE, Gowdak LHW and Matos LDNJ contributed to data analysis and interpretation. Assumpção CRA, Prado DML and Matos LDNJ wrote the manuscript. All of the authors have given final approval and agree to be responsible for all aspects of the work, ensuring accuracy and precision.

## Declaration of Competing Interest

The authors declare no conflicts of interest.
